# A novel noninvasive approach for evaluating work of breathing indices in a developmental rat model using respiratory inductance plethysmography

**DOI:** 10.1038/s41598-020-77731-6

**Published:** 2020-11-26

**Authors:** Yan Zhu, Zubair H. Aghai, Suhita Gayen Nee Betal, Michael Favara, Gina Fong, Tariq Rahman, Thomas H. Shaffer

**Affiliations:** 1 Department of Biomedical Research and Center for Pediatric Lung Research, Nemours/Alfred I. duPont Hospital for Children, P.O. Box 269, Wilmington, DE 19803 USA; 2 Pediatrics and Neonatology, Sidney Kimmel Medical College at Thomas Jefferson University, Philadelphia, PA USA; 3 Division of Neonatology, Nemours/Alfred I. duPont Hospital for Children, Wilmington, DE USA

**Keywords:** Medical research, Animal disease models

## Abstract

Pulmonary function testing (PFT) is an important component for evaluating the outcome of experimental rodent models of respiratory diseases. Respiratory inductance plethysmography (RIP) provides a noninvasive method of PFT requiring minimal cooperation. RIP measures work of breathing (WOB) indices including phase angle (Ф), percent rib cage (RC %), breaths per minute (BPM), and labored breathing index (LBI) on an iPad. The aim of this study was to evaluate the utility of a recently developed research instrument, *pneu*RIP, for evaluation of WOB indices in a developmental rat model. Sprague Dawley rats (2 months old) were commercially acquired and anaesthetised with isoflurane. The *pneu*RIP system uses two elastic bands: one band (RC) placed around the rib cage under the upper armpit and another band (AB) around the abdomen. The typical thoracoabdominal motion (TAM) plot showed the abdomen and rib cage motion in synchrony. The plots of phase angle and LBI as a function of data point number showed that values were within the range. The distribution for phase angle and LBI was within a narrow range. *pneu*RIP testing provided instantaneous PFT results. This study demonstrated the utility of RIP as a rapid, noninvasive approach for evaluating treatment interventions in the rodent model.

## Introduction

Rodent models have become the most commonly used experimental mammalian system substituting the human being in biomedical research^1,2^. Currently, 80% of experimental animals are rodents—mice, rats, and guinea pigs. Rats were most widely used in research, followed by mouse, rabbit, dog, pig, and primate^1,3^.

Pulmonary function testing (PFT) is an important component in evaluating the outcome of experimental rodent models of respiratory diseases, and it can be used to assess physiological biomarkers of underlying pulmonary disease. However, it is difficult to perform conventional PFTs on conscious rodents because of the small size and lack of cooperation. Furthermore, small animals require sensitive and rapidly responding equipment, and the signals may be small and rapid because of small animals’ anatomy and physiology^4^.

Currently, invasive and noninvasive techniques are available for the measurement of pulmonary function in mice and rats. Each technique has its advantages and disadvantages. In a review, Bates and Irvin evaluated the currently available PFTs in mice and identified a trade-off between noninvasiveness and precision. They found that noninvasive procedures such as unrestrained plethysmography necessarily sacrifice measurement precision for convenience. At the other extreme, the most precise and specific measurements resulted from the most invasive procedures, such as the forced oscillation technique, which requires mice to be anaesthetised, paralysed, and tracheostomised^5^. In safety pharmacology testing and toxicological studies, Hoymann concluded that both invasive and noninvasive pulmonary function tests provided useful and reliable information on pulmonary mechanics. Noninvasive head-out body plethysmography has been shown to be useful in measuring lung function in juvenile rats starting from day two of age^6^. Vijayaraghavan et al. used noninvasive techniques in conscious and non-anaesthetised rodents to evaluate breathing pattern and to detect sensory irritation, pulmonary irritation, and airflow limitation^7,8^.

Respiratory inductance plethysmography (RIP) and pneumotachography have been developed to measure pulmonary function in paediatric patients who are minimally cooperative^9,10^. The RIP technique uses the Respitrace system (Sensormedics, Yorba Linda, CA, USA), a cart-mounted, wired system^11^; breathing pattern and only a few parameters can be viewed on the monitor during the test, and the data analysis has to be processed off-line and is time consuming, which limits timely diagnosis and treatment decisions^11–13^. In a rat model of native airway hyper-responsiveness, at the endpoint of the study, invasive pulmonary function measurement was conducted to measure respiratory resistance and compliance on tracheostomised and mechanically ventilated mice using the plethysmography system of the plethysmography/ventilator chamber FinePointe RC (Buxco Research Systems, Wilmington, NC, USA)^14^.

Recently, our group has utilized the *pneu*RIP system (Creative Micro Designs, Newark, DE, USA), a new, noninvasive pulmonary function instrument that employs the RIP technique to test infants and children with pulmonary disorders. The hardware of this system includes a palm-sized hardware module and an iPad (Apple, Cupertino, CA, USA). The *pneu*RIP can wirelessly receive RIP data from the rodent and displays real-time work of breathing (WOB) parameters instantaneously on the iPad^15^. The WOB indices include phase angle (Ф), percent rib cage (RC %), breaths per minute (BPM), and labored breathing index (LBI). Benchtop experiments have been conducted to test the reliability, sensitivity, and reproducibility of this system^15^. Furthermore, the *pneu*RIP system was tested with 10 normal children in normal breathing conditions and with an added resistive load to compare with an existing system, Respitrace. The two systems were consistent in the measurement of phase angle, RC %, and LBI. The *pneu*RIP is more accurate than the Respitrace system for breathing rate^15^. The *pneu*RIP system and Respitrace are interchangeable in measuring the WOB indices. In a recent clinical study, our group performed *pneu*RIP testing on 9 healthy volunteers and 7 patients with Duchenne muscular dystrophy (DMD). The patients with DMD showed diffuse breathing patterns and a significant elevation in Φ, LBI, and heart rate (HR). Healthy subjects and patients with DMD had similar BPM and RC % averages. A mean ± standard error of the mean (SEM) of phase angle of 23.40° ± 4.33° and LBI of 1.07 ± 0.02 were reported in healthy subjects^16^.

While the normal values of pulmonary function tests in humans who are conscious and cooperative are well established, little is known regarding pulmonary function values such as WOB indices in anaesthetised rodents. This study is the first to use this newly developed RIP technique, *pneu*RIP, on rodents. As such, the aim of this feasibility study was to evaluate the utility of the developed research instrument, *pneu*RIP, for evaluation of WOB indices in a developmental rat model.

## Materials and methods

### Animal preparation

This study was approved by the Institutional Animal Care and Use Committee (IACUC) of Nemours/Alfred I. duPont Hospital for Children, and all methods were performed in accordance with the National Institutes of Health’s Guide for the Care and Use of Laboratory Animals. This pilot study was conducted with 5 Sprague Dawley rats (245.9 ± 55.8 g, 2 months old) that were commercially acquired (Charles River Laboratories, Malvern, PA, USA). Rats were anaesthetised for 3 min with an induction dose of 5% isoflurane vapor with 40% fraction of inspired oxygen (FiO_2_) and maintained with 3% inhaled isoflurane with 40% FiO_2_. The rats were placed in a right lateral lying position on an infrared warming pad (Kent Scientific Corporation, Torrington, CT, USA) during 3.5 min of testing. Oxygen saturation and heart rate were monitored using a pulse oximeter (MouseSTAT, Smiths Medical, Waukesha, WI, USA). End-tidal carbon dioxide (ETCO_2_) was measured using a Non-invasive Cardiac Output (NICO) monitor (Novametrix Medical Systems, Wallingford, CT, USA).

### pneuRIP measurement

**Figure 1:**
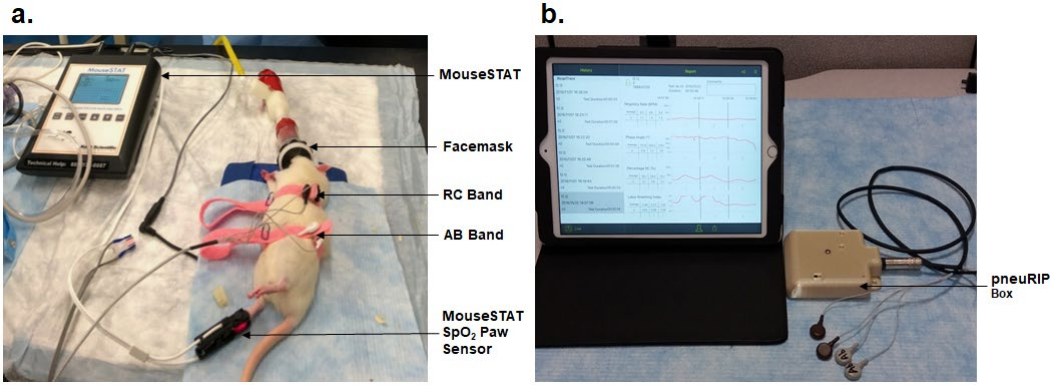
(**a**) Respiratory inductance plethysmography using the *pneu*RIP. The *pneu*RIP system uses two elastic bands: one band (RC) placed around the rib cage under the upper armpit and another band (AB) around the abdomen. (**b**) The *pneu*RIP wirelessly connects to the iPad and displays data in real-time plots.

Respiratory inductance plethysmography using the *pneu*RIP (hardware and software provided by Creative Micro Designs, Newark, DE, USA) was conducted on the 5 Sprague Dawley rats. The *pneu*RIP system uses two elastic bands embedded with insulated induction wires, which measure the movement and synchrony of the rat’s rib cage and diaphragm^17^; one band (RC) is placed around the rib cage under the armpit of the upper limb and another band (AB) is placed around the abdomen at the largest abdominal circumference parallel to the RC band (Fig. 1a).

The *pneu*RIP wirelessly connects with the iPad to plot the data points and calculates the RIP parameters (Fig. 1b). During 3.5 min of testing, movements of the rib cage and abdomen were plotted, and WOB indices were calculated and continually recorded in real time, which included LBI, Ф between abdomen and rib cage, respiratory rate, and RC %^15,16^.

As previously described^15,16^, WOB indices are defined as follows:

1. Percent rib cage (RC %), which is a ratio of the absolute magnitude of RC signal to the sum of the absolute magnitudes of the RC and ABD signals. The RC %, shown in Eq. (1), defines RC contribution to tidal volume and is presented as a percent of the combined RC and ABD volume changes.
  1 $$RC \%=\frac{\left|RC\right|}{\left|RC\right|+\left|ABD\right|}$$
  where $\left|RC\right|$ is the magnitude of the signal recorded from the rib cage and $\left|ABD\right|$ is the magnitude of the signal recorded from the abdominal compartment.
2. Labored breathing index (LBI) (Eq. 2), which is a measure of respiratory effort due to asynchronous breathing. LBI is a ratio that is an estimate of WOB effort.
  2 $$LBI=\frac{{\left|RC\right|}_{t}^{inphase}+{\left|ABD\right|}_{t}^{inphase}}{{\left|RC\right|}_{t}+{\left|ABD\right|}_{t}}$$
  where ${\left|RC\right|}_{t}$ and ${\left|ABD\right|}_{t}$ are the magnitudes of the RC and ABD compartment signals at any instant of time (t), while ${\left|RC\right|}_{t}^{inphase}$ and ${\left|ABD\right|}_{t}^{inphase}$ are the magnitudes when the signals are in-phase. The denominator is the tidal volume (V_T_), which is the sum of the absolute values of the RC and ABD signals, as recorded. The numerator is the maximum compartmental volume and is the sum of the absolute values of the RC and ABD signals when in unison.
3. Phase difference (ϕ), which is a measure of synchrony between RC and ABD compartments. The phase angle (Φ) between the RC and ABD is calculated by normalizing the signals over 20 samples. The $\phi$ is then calculated by Eq. (3), expressed as follows:
  3 $$\phi ={\mathrm{cos}}^{-1}\frac{\sum_{\mathrm{n}=0}^{\mathrm{N}-1}\mathrm{x}\left(\mathrm{n}\right)\mathrm{y}\left(\mathrm{n}\right)}{\sqrt{\left[\sum_{\mathrm{n}=0}^{\mathrm{N}-1}{\mathrm{x}\left(\mathrm{n}\right)}^{2}\sum_{\mathrm{n}=0}^{\mathrm{N}-1}{\mathrm{y}\left(\mathrm{n}\right)}^{2}\right]}}$$
  where x represents RC, y represents ABD, and n is the number of samples.
4. Respiratory rate in breaths per minute. This was determined by a fast Fourier transform (FFT) algorithm to generate the average magnitudes for each frequency of the RC and ABD signal. From these calculations, an array is generated from which the largest value is selected, as well as amplitudes within 20% of this value. Finally, we compute a weighted average using indices of these values. This value is the frequency multiplied by 60 to obtain breaths per minute.

During the RIP measurements, patterns of breathing were determined by recording the signals of the rib cage and abdomen. After the test was completed, the data recordings were securely saved on the iPad and were transferred via secured email for further analysis. Breaths per minute (BPM) were calculated at a rate of 10 measurements per second.

### Statistical analysis

Statistical software SPSS version 25 (IBM, Armonk, NY, USA; https://www.ibm.com/products/spss-statistics) and Excel 2016 (Microsoft, Redmond, WA, USA) were used to perform the statistical analysis and data plotting. The pulmonary function summary data were presented in tabular form as mean and SEM (Table 1). To date, there have been little data reported on RIP in rats, so we compared our rat data with other RIP data from human infants and children. Typical plots of Ф and LBI were made as a function of data point number (10 points per second) in an individual rat and data collection period. In addition, the overall distribution histograms for all five rats were plotted to identify the distribution of group breathing patterns over time and for comparison with previous studies.

**Table 1:** Physiologic and pulmonary function summary (mean ± SEM).

	Weight (g)	HR (beats/min)	SPO_2_ (%)	ETCO_2_ (mmHg)	BPM (breaths/min)	Phase angle (°)	RC %	LBI
Mean	245.9	345.2	95.8	27.2	56.6	8.94	53.19	1.04
SEM	24.94	10.30	0.73	0.97	5.06	0.22	0.19	0.001

*SEM* standard error of the mean, *HR* heart rate, *SPO*_*2*_ saturation of peripheral oxygen, *ETCO*_*2*_ end-tidal carbon dioxide, *BPM* breaths per minute, *RC %* percent rib cage, *LBI* labored breathing index.

## Results

The mean and SEM of physiological and pulmonary function parameters are summarized in Table 1. The variables include the WOB indices (Ф, RC %, BPM, LBI), HR, weight, saturation of peripheral oxygen (SPO_2_), and ETCO_2_. As shown, mean WOB indices, Ф = 8.94°, RC % = 53.19, and LBI = 1.04, were in very narrow ranges as indicated by their respective SEM values. Likewise, the physiological results (mean ± SEM) of HR = 345.2 ± 10.30 beats/min, SPO_2_ = 95.8 ± 0.73%, ETCO_2_ = 27.2 ± 0.97 mmHg, and BPM = 56.6 ± 5.06 breaths/min were within a very tight range as indicated by SEM values and, in general, within expected normal physiological ranges.

**Figure 2:**
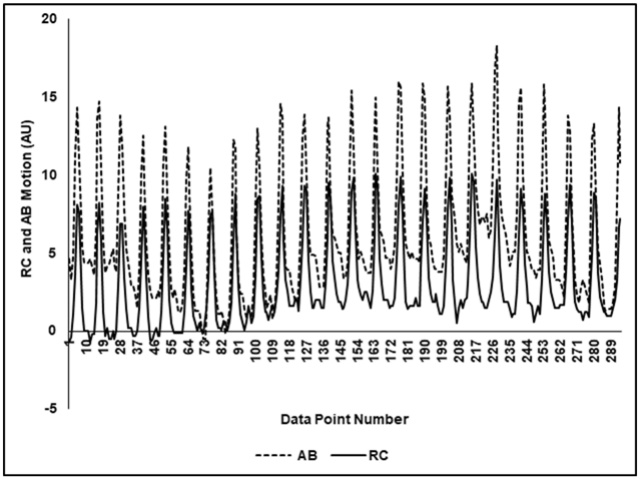
A typical plot of the thoracoabdominal motion (TAM) as a function of sample number/time. As illustrated, the abdomen (AB) and rib cage (RC) motion were of similar amplitude and almost superimposed as a function of sample number/time. The plot showed the abdomen and rib cage motion both in synchrony.

Figure 2 shows a typical plot of the thoracoabdominal motion (TAM) as a function of sample number/time in the same rat and time segment. As illustrated, the abdomen and rib cage motion were of similar amplitude and almost superimposed as a function of sample number/time, thus indicating a synchronous breathing pattern consistent with a low mean phase angle of 4.42° and mean LBI of 1.001.

**Figure 3:**
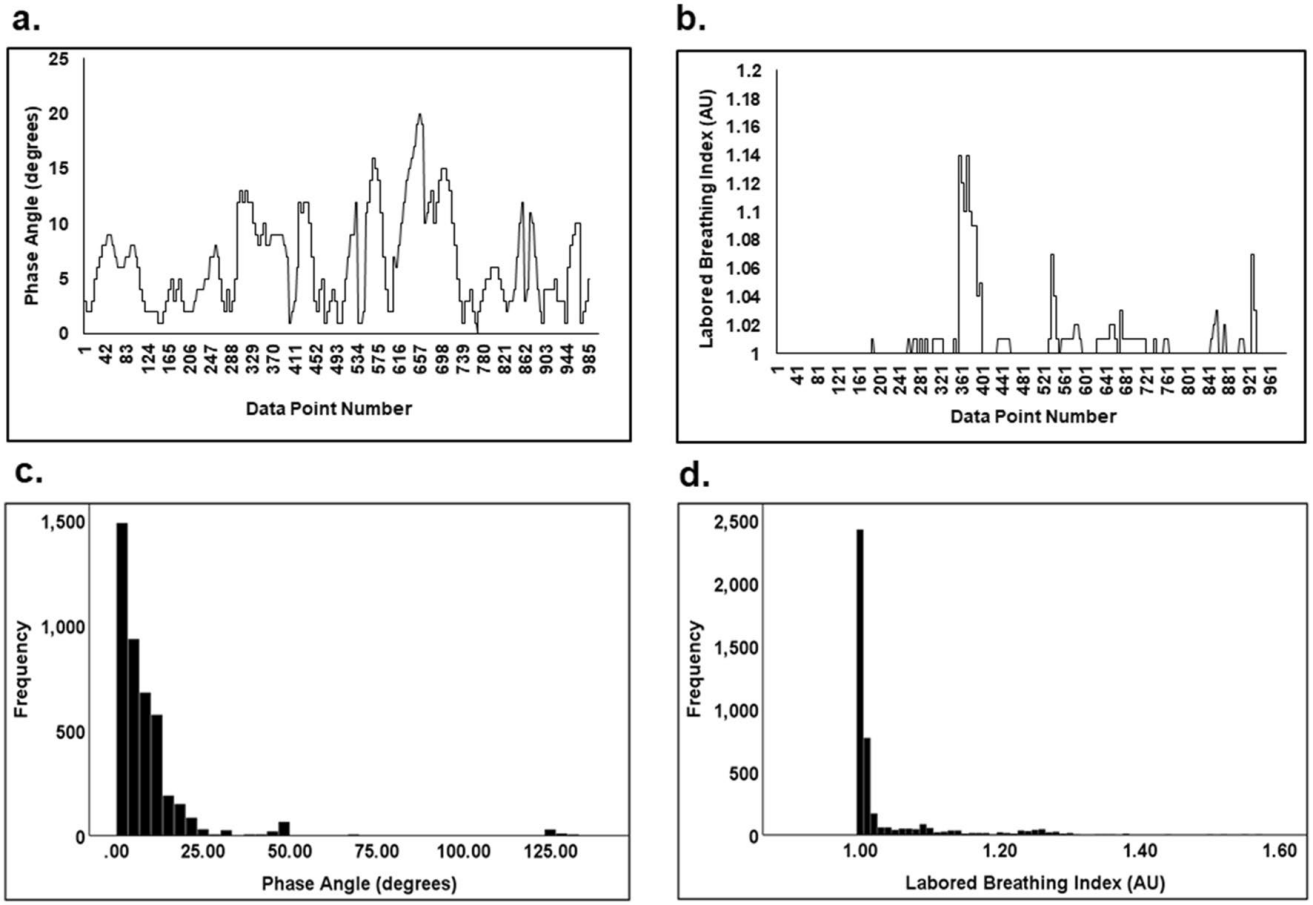
Typical plots of phase angle (Ф) and LBI as a function of data point number/time in an individual rat during the same recording segment as shown in (**a**) and (**b**): (**a**) The phase angle values were within the range (0 ≤ Ф ≤ 20°); (**b**) The LBI values were within the range (1 ≤ Ф ≤ 1.16). Histograms of five rat data in phase angle and LBI as shown in (**c**) and (**d**): (**c**) Phase angle histogram of five rat data: 96.2% of the data values were within the range (0 ≤ φ ≤ 30°) with a mean ± SEM of 8.94 ± 0.22; (**d**) LBI histogram of five rat data: 88.5% of the data values were within the range with a mean ± SEM of 1.04 ± 0.001.

With regard to WOB indices, most noteworthy are the means and distribution of phase angle and LBI over the study duration^11,12,15,16^. As shown in Fig. 3, typical measurements of phase angle and LBI of the same rat and time segment are plotted as a function of data point number/time over the study duration. As illustrated in Fig. 3a, the phase angle values were within the range (0 ≤ Ф ≤ 20°). As shown in Fig. 3b, the LBI values were within the range (1 ≤ Ф ≤ 1.16).

Phase angle and LBI group histograms of all 5 rats are plotted in Fig. 3c and d to demonstrate how these RIP parameters are distributed as a function of measurement range. The phase angle histogram (Fig. 3c) shows a clustering of measured data points below 30° and demonstrates that 96.2% of the phase angle data values were within the range (0 ≤ Ф ≤ 30°). As shown in Fig. 3d, the LBI histogram shows a clustering of measured data points between 1 and 1.10; the LBI histogram demonstrates that 88.5% of the data values were within the range (1.00 < LBI < 1.10).

## Discussion

In this study, noninvasive *pneu*RIP testing was performed on anaesthetised, spontaneously breathing, 2-month-old rats. The primary objective was to demonstrate the utility of the newly developed *pneu*RIP system for evaluation of WOB indices including Ф, RC %, BPM, and LBI for developing rats.

The relationship between age of laboratory rats and human has been established^1,2^. Rats become sexually mature at about 6 weeks old. In adulthood, every month of the animal is approximately equivalent to 2.5 human years^1^. The rats used for the current study were 2 months old, which is equivalent to young adult in humans^2^. In most cases, the PFT changes in small animals correspond to changes in human PFTs^4^. In general, for humans and most mammals, normal synchronous breathing is observed at Ф < 30° and LBI < 1.05^18^. A phase angle of 0° represents perfect synchrony between the chest and abdominal compartments^19^. Higher phase angles represent greater degrees of asynchronous breathing, and a phase angle of 180° represents paradoxical breathing^13^. The LBI is an indicator of the additional WOB required to maintain gas exchange when breathing is asynchronous and is associated with pulmonary dysfunction^10–12,16,17^.

As shown in Table 1, the mean ± SEM of phase angle of 8.94 ± 0.22° and LBI of 1.04 ± 0.001 for rats were considered within the normal range compared with humans based on our previous studies^15,17,18^ as well as others^10–12^. The results of HR, SPO_2_, ETCO_2_, and BPM were consistent with the results in our previous rat study^14^.

As shown in Fig. 2, the typical TAM plot showed the abdomen and rib cage motion both in synchrony, which was similar to the pattern of real-time plots displayed on the iPad in previous healthy human studies^10–12,15,16^. Figure 3a and b illustrate typical plots of phase angle and LBI as a function of data point number and show that the values of phase angle and LBI were within the range (0 ≤ Ф ≤ 20°, 1 ≤ LBI ≤ 1.16, respectively). These data demonstrated the study rats had synchronous breathing and, coupled with normal cardiopulmonary measurements (Table 1), indicated no pulmonary dysfunction. As shown in Fig. 3c and d, data from all five rats were plotted as histograms indicating data distribution over the study duration. The phase angle histogram (Fig. 3c) showed a clustering of measured data points below 30° and demonstrated that 96.2% of the phase angle data values were within the range (0 ≤ Ф ≤ 30°) as shown for healthy humans^15,18^. In Fig. 3d, the LBI histogram showed a clustering of measured data points between 1 and 1.10; the LBI histogram demonstrated that 88.5% of the data values were within the normal range. The distribution for phase angle and LBI was within a narrow normal range, a single-distribution clustering^15,18^. These WOB index findings were consistent with those of our previous human studies^12,16,17^.

Warren et al. performed a semi-quantitatively calibrated, noninvasive RIP on 61 preterm infants and 7 full-term infants. Mean (± SD) phase angle was significantly greater in preterm infants than in full-term infants (60.6 ± 39.8 versus 12.5 ± 5.0). Mean (± SD) LBI was significantly greater in preterm infants than in full-term infants (1.35 ± 0.35 versus 1.01 ± 0.01). The rib cage contribution to breathing did not differ significantly between preterm and full-term infants^13^. The phase angle and LBI data of full-term infants support the findings of our current study, in which phase angle and LBI were the key measurements of synchrony related to respiratory function.

Chest wall motion (CWM) was evaluated using RIP before and during mechanical ventilation to evaluate five children and young adults with neuromuscular (NM) disease. It was shown that mechanical ventilation improved RC/ABD asynchrony and decreased Φ in this NM patient population^20^. As shown, the authors concluded that assessment of CWM with RIP may be useful in evaluating respiratory function in an intensive care setting, as well as assisting in the determination of optimal ventilator settings in NM patients.

Over the years, RIP measurements have been utilized in determining differences between healthy subjects and patients with respiratory complications using post-hoc analysis with Respitrace. In this regard, Giordano et al.^10^ showed that RIP data correlated with admission criteria in pediatric asthmatic patients in the emergency department. They found that Φ, LBI, and HR were all elevated in those patients who were admitted to the hospital compared with those who were discharged to home. Furthermore, Rodriguez et al.^21^ demonstrated that RIP measurements could identify thoracic problems in children with skeletal dysplasia. Another skeletal dysplasia study demonstrated the utility of RIP in Morquio patients in combination with spirometry and forced oscillation techniques^22^. Kubaski et al. noted in a large population of Morquio patients 3 to 40 years of age that these patients had normal breathing synchrony using combined RIP and impulse oscillometry technology. Although these patients had small lung volumes due to Morquio syndrome, they exhibited no clinical or functional signs of restrictive or obstructive lung disease. It is noteworthy that RIP was essential in identifying that patients with Morquio have small lungs with synchronized breathing while maintaining normal gas exchange at rest^22^.

### Study limitations

Noninvasive *pneu*RIP testing provides instantaneous PFT results requiring minimal patient cooperation as previously noted above. However, rodents need to be anaesthetised with either inhalant gas or injectable drugs during the measurement process. The use of inhalant gas is the preferred method of anaesthesia whenever possible. During testing, it is important to control the depth of anaesthesia and keep constant positioning across measurements and the same anaesthesia level for each rat. If rats are exposed to excessive isoflurane, the rats’ breathing would become shallow and irregular, and some signals of the TAM would become too weak to be detected by the *pneu*RIP software. In this study, it should be noted that the rat sample size was limited; thus, more normal rats would be necessary to measure to represent the population values for all rats. With regard to repeatability and credibility of the *pneu*RIP system, it has been validated with respect to bench studies and healthy volunteer comparisons with the gold standard, Respitrace^15^.

It is noteworthy to mention that the variations in WOB indices (phase angle and LBI) are quite small and that the normality of phase angle and LBI for the presented rodents is similar to that reported for healthy humans in the age range of 3 to 18 years^15,17,18^.

### Conclusion

The *pneu*RIP testing provided instantaneous PFT results for rapid and noninvasive study of rodent breathing patterns and WOB indices. The *pneu*RIP method was precise and accurate in measuring the phase angle, RC %, and LBI. This novel approach for assessment of PFT translated appropriately to a small experimental rodent model, and, in addition to providing immediate results, the method has the potential for studying longitudinal PFT outcomes in a developing rat model. As previously noted, this experimental model has been used to study many respiratory neonatal disorders such as hyperoxia, barotrauma, sepsis, etc.

## Data Availability

The datasets generated during and/or analysed during the current study are available from the corresponding author on reasonable request.

## References

[1] Andreollo NA, Santos EF, Araujo MR, Lopes LR (2012). Rat’s age versus human’s age: what is the relationship?. Arq. Bras. Cir. Dig..

[2] Sengupta P (2013). The laboratory rat: relating its age with human’s. Int. J. Prev. Med..

[3] Vandamme TF (2014). Use of rodents as models of human diseases. J. Pharm. Bioallied Sci..

[4] Whitehead, G. S., Kimmel, E. C., Reboulet, J. E. & Still, K. R. Pulmonary function in normal rats. Naval Health Research Center Detachment (Toxicology), Report No. TOXDET 99–5 (1999).

[5] Bates JH, Irvin CG (2003). Measuring lung function in mice: the phenotyping uncertainty principle. J. Appl. Physiol..

[6] Hoymann HG (2012). Lung function measurements in rodents in safety pharmacology studies. Front. Pharmacol..

[7] Vijayaraghavan R, Schaper M, Thompson R, Stock MF, Alarie Y (1993). Characteristic modifications of the breathing pattern of mice to evaluate the effects of airborne chemicals on the respiratory tract. Arch. Toxicol..

[8] Vijayaraghavan R (1994). Computer assisted recognition and quantitation of the effects of airborne chemicals acting at different areas of the respiratory tract in mice. Arch. Toxicol..

[9] Bhatia R (2011). Surfactant administration prior to one lung ventilation: physiological and inflammatory correlates in a piglet model. Pediatr. Pulmonol..

[10] Giordano K (2012). Pulmonary function tests in emergency department pediatric patients with acute wheezing/asthma exacerbation. Pulm. Med..

[11] Allen JL (1991). Interaction between chest wall motion and lung mechanics in normal infants and infants with bronchopulmonary dysplasia. Pediatr. Pulmonol..

[12] de Jongh BE (2014). Work of breathing indices in infants with respiratory insufficiency receiving high-flow nasal cannula and nasal continuous positive airway pressure. J. Perinatol..

[13] Warren RH, Horan SM, Robertson PK (1997). Chest wall motion in preterm infants using respiratory inductive plethysmography. Eur. Respir. J..

[14] Rodriguez E (2014). The orl rat is more responsive to methacholine challenge than wild type. Pulm. Pharmacol. Ther..

[15] Rahman T (2017). *pneu*RIP: a novel respiratory inductance plethysmography monitor. J. Med. Device..

[16] Ryan L, Rahman T, Strang A, Heinle R, Shaffer TH (2020). Diagnostic differences in respiratory breathing patterns and work of breathing indices in children with Duchenne muscular dystrophy. PLoS ONE.

[17] Strang A (2018). Measures of respiratory inductance plethysmography (RIP) in children with neuromuscular disease. Pediatr. Pulmonol..

[18] Balasubramaniam SL (2019). Age-related ranges of respiratory inductance plethysmography (RIP) reference values for infants and children. Paediatr. Respir. Rev..

[19] Chadha TS (1982). Validation of respiratory inductive plethysmography using different calibration procedures. Am. Rev. Respir. Dis..

[20] Diaz CE, Deoras KS, Allen JL (1993). Chest wall motion before and during mechanical ventilation in children with neuromuscular disease. Pediatr. Pulmonol..

[21] Rodriguez ME (2010). Skeletal dysplasias: evaluation with impulse oscillometry and thoracoabdominal motion analysis. Pediatr. Pulmonol..

[22] Kubaski F (2015). Non-invasive pulmonary function test on Morquio patients. Mol. Genet. Metab..

